# Manufacturing of PEEK orthodontic baseplate and 3D-printed alloy components from an intraoral scan

**DOI:** 10.1097/MD.0000000000038004

**Published:** 2024-04-26

**Authors:** Noor A. Al Mortadi, Lina Khasawneh, Karem H. Alzoubi

**Affiliations:** a Department of Applied Dental Sciences, Faculty of Applied Medical Sciences, Jordan University of Science and Technology, Irbid, Jordan; b Department of Prosthodontics, Faculty of Dentistry, Jordan University of Science and Technology, Irbid, Jordan; c Department of Pharmacy Practice and Pharmacotherapeutics, University of Sharjah, Sharjah, United Arab Emirates; d Department of Clinical Pharmacy, Faculty of Pharmacy, Jordan University of Science and Technology, Irbid, Jordan.

**Keywords:** 3D printed alloy, intraoral scan, manufacturing, PEEK orthodontic baseplate

## Abstract

This paper demonstrates a digital manufacturing technique of a removable orthodontic appliance from an intraoral scan. An intraoral scan was made for the maxillary and mandibular arches. 3Shape Orthodontics Appliance Designer produced the virtual Hawley retainer, consisting of alloy components (Adam Clasps and Fitted Labial bow) and a base plate. The base plate design was modified to adapt to inserting the alloy components, which were combined using cold-cured acrylic. The finished Hawley retainer was assessed intraorally. The described technique emphasizes the design specifications of digitally designed and manufactured removable orthodontic appliances. A combination of additive and subtractive techniques was successfully employed to manufacture the alloy components and base plate. This novel method provides an alternative approach to manufacturing removable appliances with computer-aided design (CAD)/computer-aided manufacturing (CAM) technologies. The described process offers a precursor to digital manufacturing of other developed designs of dental appliances.

## 1. Introduction

Digital technology has been increasingly adopted in designing and fabricating various types of removable appliances.^[1,2]^ The user-friendly interface of the specialized Computer-aided design/computer-aided manufacturing (CAD/CAM) systems, in addition to the revolution of intraoral scanners, enables the adoption of novel designs to manufacture prostheses entirely digitally without the need to make conventional impressions.^[1,2]^

Several specialized orthodontic digital design software programs have been developed, such as the Invisalign System (Align Technology, Inc., Santa Clara, CA), which is used to build clear, customized aligners, and the Incognito system (3M-Unitek Monrovia, Calif), which produces direct 3-dimensionally printed (3DP) customized lingual brackets.^[3–5]^

Additive manufacturing (AM), also known as 3D printing or rapid prototyping, is a process of creating objects by joining materials layer by layer, as defined by the American Society for Testing and Materials.^[6]^ This technology has been integrated into digital manufacturing hardware and can produce prototypes of complex structures using materials such as plastic or metals in a layered fashion. The application of AM technology in dentistry has the potential to revolutionize the economical production of dental devices, although the cost remains high due to specialized materials and equipment.^[7]^ AM has overcome the limitations of subtractive manufacturing techniques, which are unsuitable for producing complex structures with thin walls and flexible curved shapes.^[7]^ Moreover, milling complex structures in an alloy requires additional methods to fix and brace the piece to a work bed to prevent flexure during manufacturing. AM technologies gained popularity in dentistry, where material waste was reduced by 40% and enabled the creation of complex designs.^[7]^ A wide variety of machines and materials can be used at a reasonable cost.^[7]^ 3D printing in dentistry made it easier by personalizing and simplifying the complexity of prostheses and appliance production.^[1,2]^

This paper demonstrates the manufacturing method of a digitally designed and manufactured Hawley retainer from the intraoral scan. The Hawley retainer was the appliance of choice due to its design simplicity. It comprises a maxillary acrylic baseplate with a labial bow and 2 Adam clasps.

## 2. Materials and methods

### 2.1. Workflow

**Figure 1.**
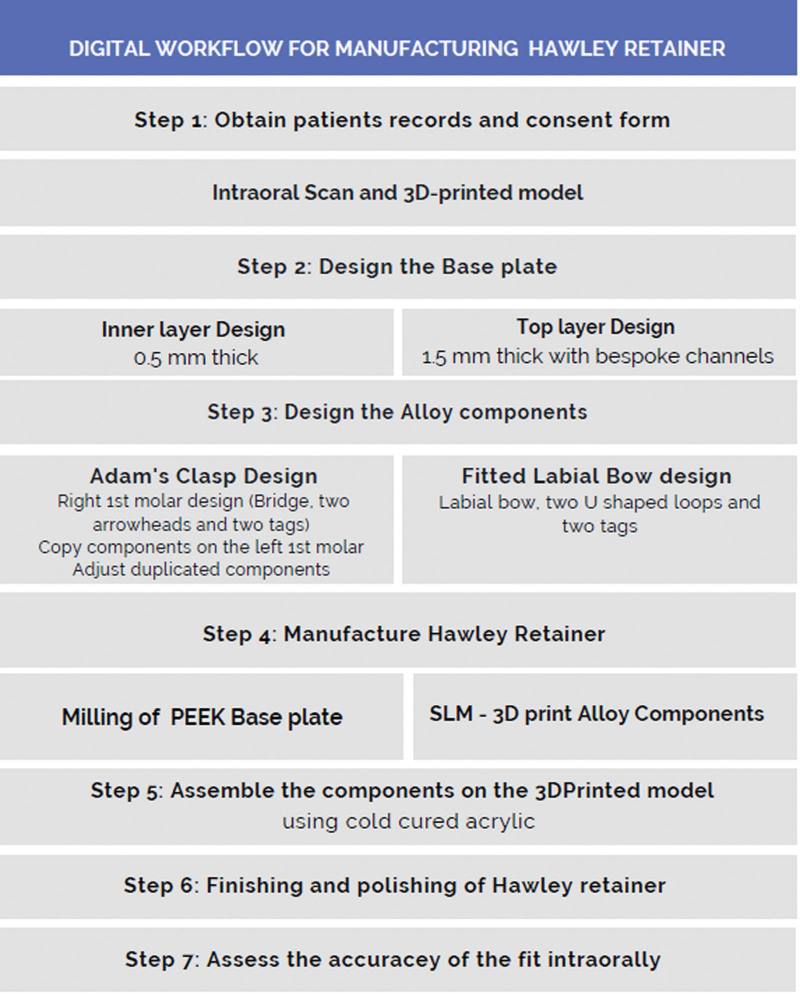
The CAD/CAM workflow. CAD = computer-aided design, CAM = computer-aided manufacturing.

A digital scan of maxillary and mandibular teeth and surrounding mucosa was taken using an intraoral scanner (3Shape intraoral scanner/TRIOS, Copenhagen, Denmark). The stereo lithography (STL) file was imported to 3Shape Orthodontics Appliance Designer software (Copenhagen, Denmark). The 3D scan was surveyed electronically to determine the insertion path and block out the undesired undercuts using the “set up insertion direction” and “create/remove undercuts” tools. 3D scanned data were exported as a STL file for resin 3D printing using a 3D printer (Next Dent Model Ortho, 3d system). Figure 1 summarizes the design and manufacturing process.

### 2.2. A summary of producing Hawley retainer

**Figure 2.**
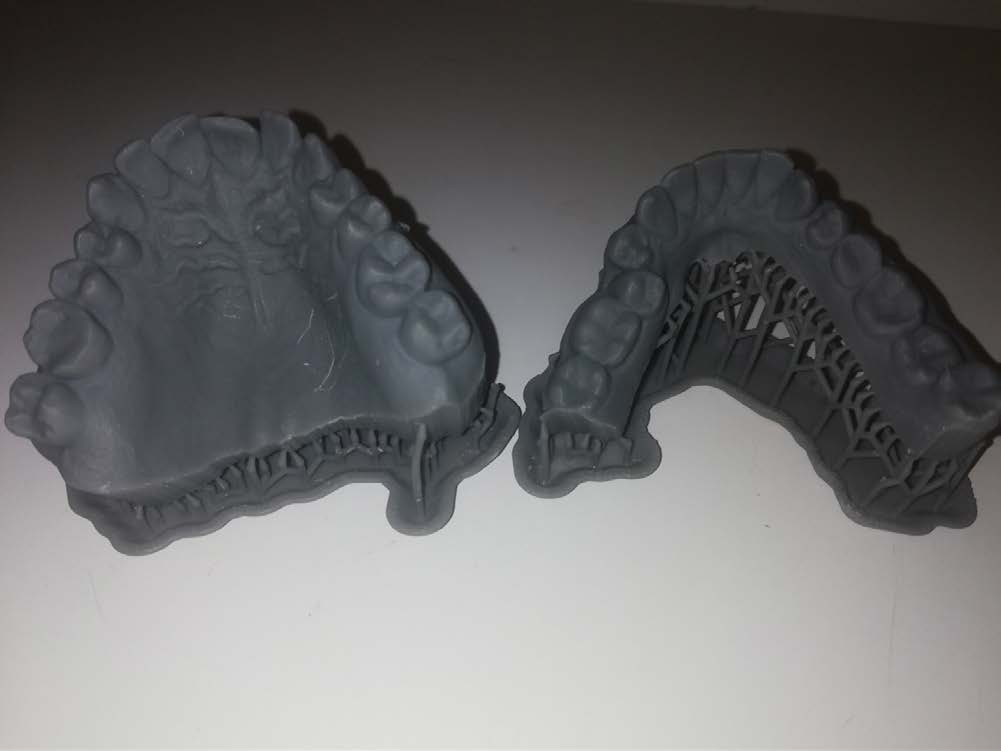
The 3D printed models for the intraoral scans.

The base plate was produced in 2 layers: an inner layer that fits directly on the surface of the scan and a top layer. The latter represents the polished surface of the base plate and has bespoke channels where the clasp tags will be inserted. The clasps were built virtually, and their tags were to be inserted in the bespoke channels. Then, the physical components were assembled with the base plate using cold-cured acrylic resin. 3D printed models for the intraoral scans were created to assist in assembling the components of the device (Fig. 2).

### 2.3. Base plate

#### 2.3.1. The inner layer of the base plate

**Figure 3.**
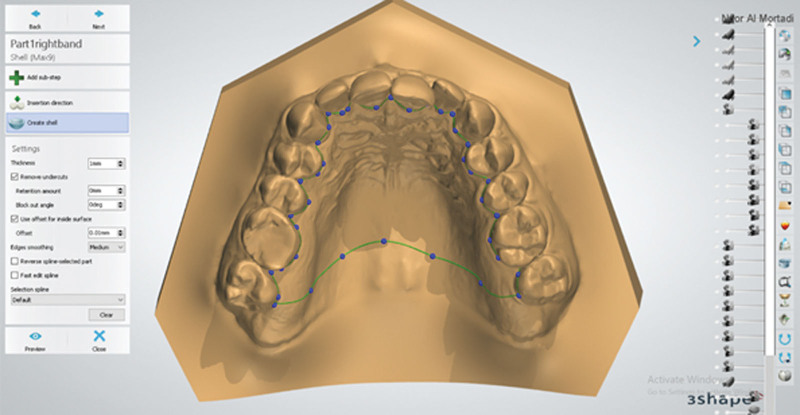
The extension outlines of the fitted layer of the base plate using 3shape.

This layer was produced by drawing a line representing the extension of base plate borders. The borders were drawn by a series of points (Fig. 3). The base plate covered one-third of the lingual surfaces of the teeth at the survey line and extended posteriorly to the distal surface of the second molars. The thickness of this layer was 0.5 mm, representing the minimum recommended space between the alloy components of clasp tags and the palate.

#### 2.3.2. The top layer of the base plate

**Figure 4.**
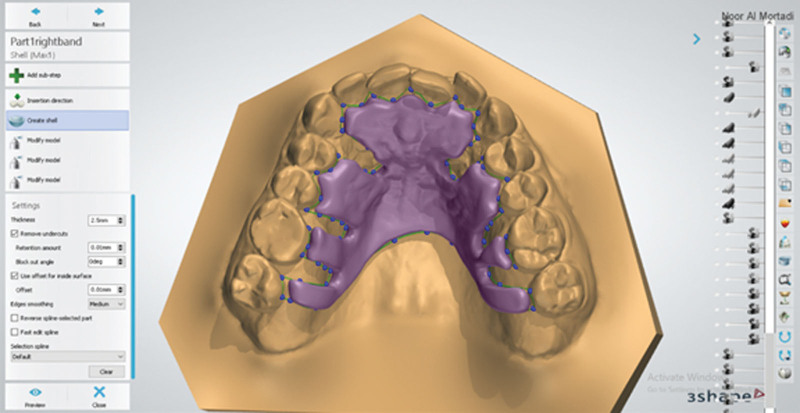
The extension outlines the outer layer of the base plate using 3shape.

**Figure 5.**
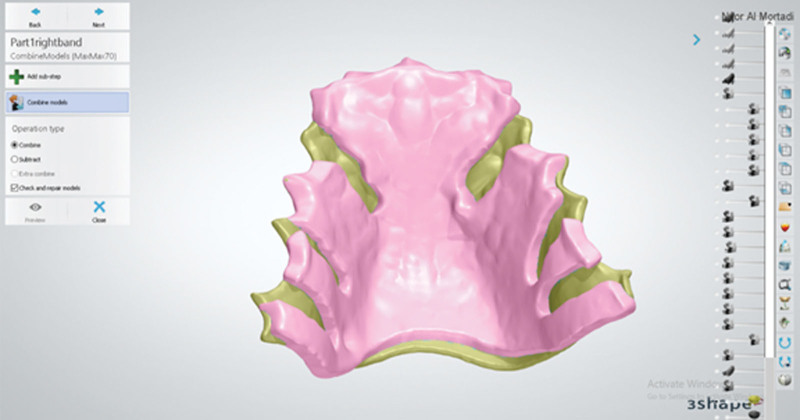
The combined fitted layer and the outer layer of the base plate.

**Figure 6.**
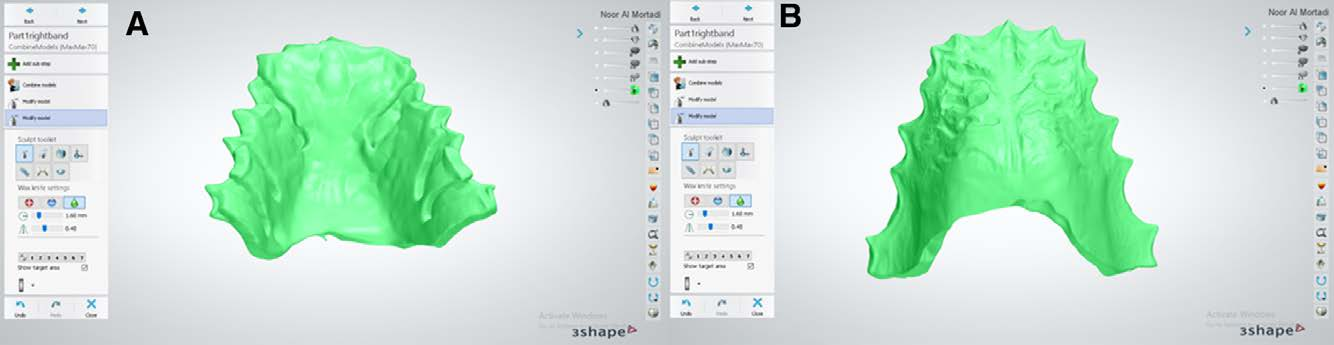
(A) Using 3Shape software the combined fitted layer and outer layer of the base plate. (B) Using 3Shape software, the fitting surface of the combined base plate.

The outline of the top layer followed the outline of the inner layer. Bespoke channels, where the clasp tags will be inserted, were cut back from the outline (Fig. 4). This layer was 1.5 mm thick. The inner and top layers were combined using the “combine models” tool (Fig. 5). The definitive baseplate is shown in Figure 6A and B.

### 2.4. Alloy components

“Bar” and “circular” cross sections of 1.0 mm diameter were selected from the control panel. The clasps were drawn on the blocked-out scan by creating a series of points to form a line. This line represents the center of the circular bar of the clasp. The software enables adjusting the position of a specific point or a group of points by moving them in any direction.

### 2.5. Adams clasps

**Figure 7.**
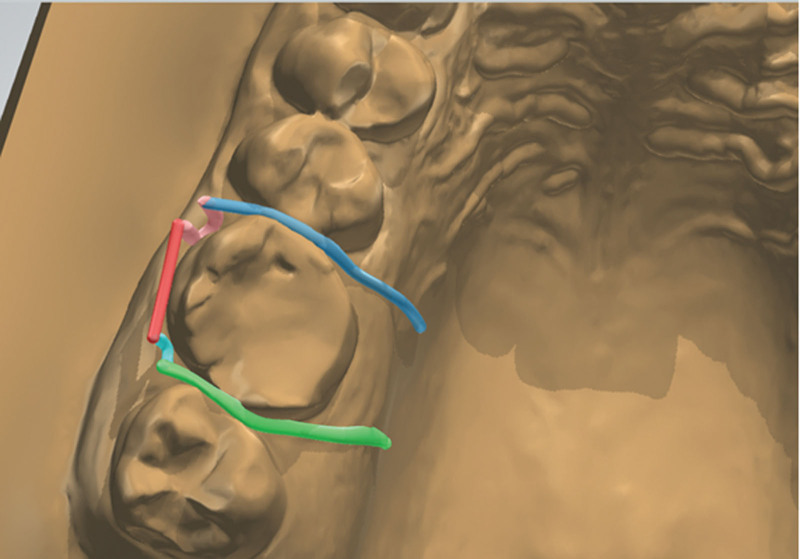
Components of upper right Adams clasp “virtual.”

Five parts were produced to construct an Adams clasp for the right first molar. A bridge, 2 arrowheads, and 2 tags. Adjacent parts were connected using the “combine models” tool (Fig. 7). Areas of connection can be thickened and smoothened using the “modify model” tool. A measuring tool was used to ensure 45 degrees between the bridge part of the clasp and the buccal surface of the tooth. The angle between the bridge and arrowheads was 45 degrees.

**Figure 8.**
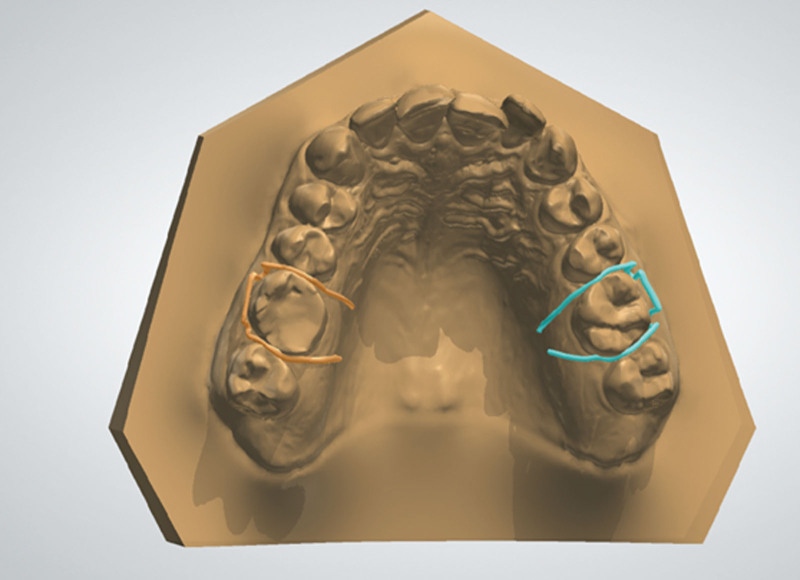
Virtual Adams clasps on the right and left first molars.

To produce an Adams clasp on the left first molar, “copy and drag” tools were used to duplicate the right molar Adams clasp components. Adjustments and alterations of the duplicate components were carried out, such as shortening the bridge or the arrowheads and changing the angulation between components. Finally, the parts on the left of the first molar were combined as described above (Fig. 8). Adams clasps were checked when the lower arch occludes against the upper arch to ensure no occlusal interference.

To ensure that the tags’ position fits accurately in the bespoke channels, the “hide” tool was used to make the top layer of the baseplate transparent to ensure no overlap between the alloy components’ tag and the inner layer of the baseplate.

### 2.6. Labial bow

The labial bow was constructed by designing 5 parts: a bow, 2 U-shaped loops, and 2 tags. The bow fits on the labial surface of the anterior teeth from the right maxillary canine to the left maxillary canine at a level of 2-thirds of the incisor-cervical length. Two U-shaped loops were drawn from the middle of the canine to the distal surface. They were made 1 mm away from the canines to prevent gingival irritation. In addition, 2 tags were built to fit in the bespoke channels. All components were joined using the “combine models” tool described previously.

#### 2.6.1. The physical build of components of Hawley retainer

**Figure 9.**
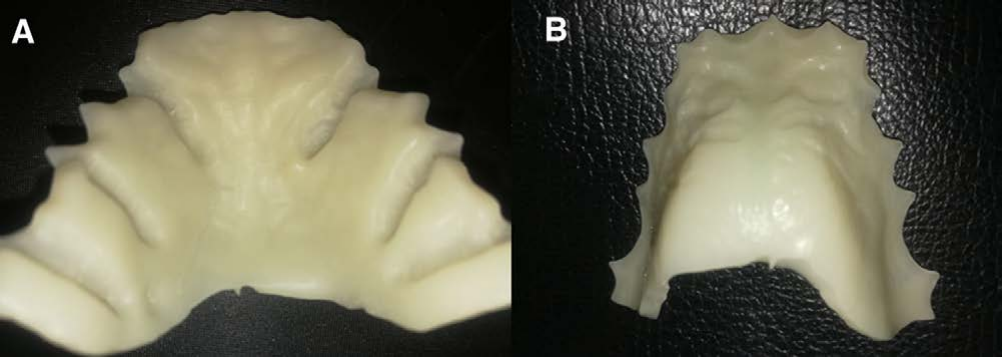
(A) The finishing surface of the combined base plate in PEEK. (B) The fitting surface of the PEEK base plate. PEEK = polyether ether ketone.

The virtual components of the Hawley retainer were exported as STL files for manufacturing. The base plate was exported to the milling machine (Calidia WP1–5-Axis CNC-Milling) from the biocompatible PEEK material (CopraPeek high-performance resin Blank 98 mm Ø from 15 mm with step) (Fig. 9A and B).

**Figure 10.**
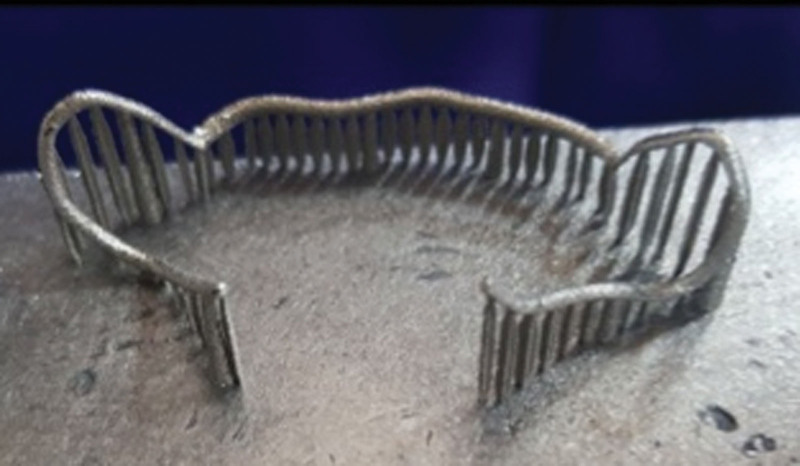
The 3D printed labial bow with the support in place.

**Figure 11.**
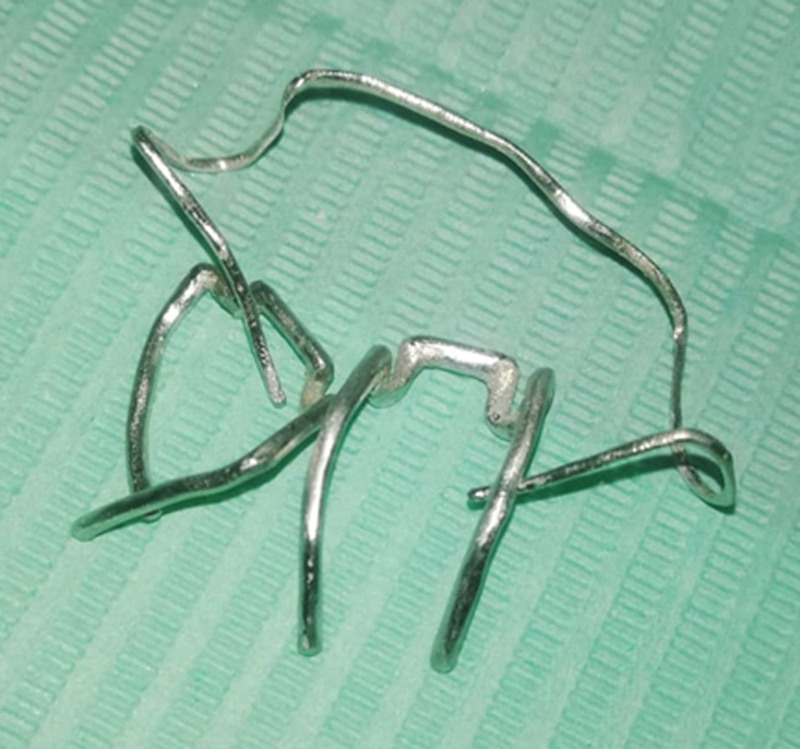
The SLM clasps after finishing. SLM = selective laser melting.

**Figure 12.**
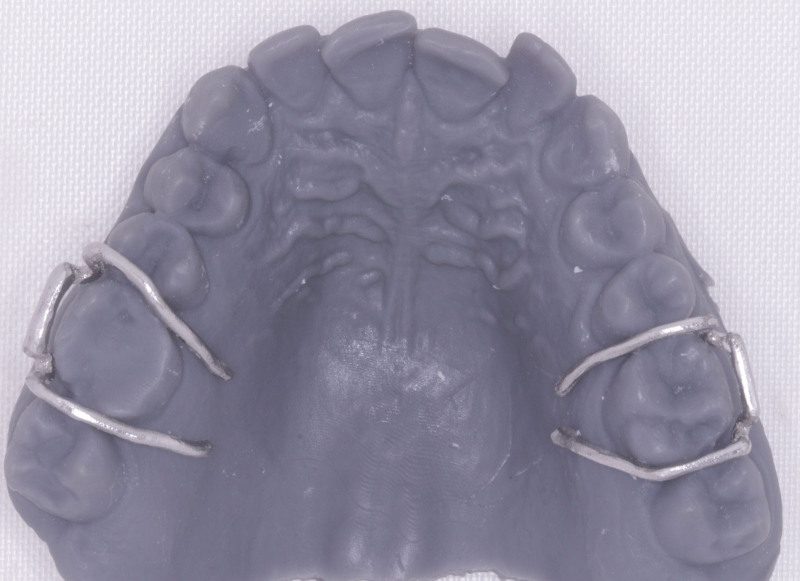
The Adams clasp fitted on 3D printed model.

**Figure 13.**
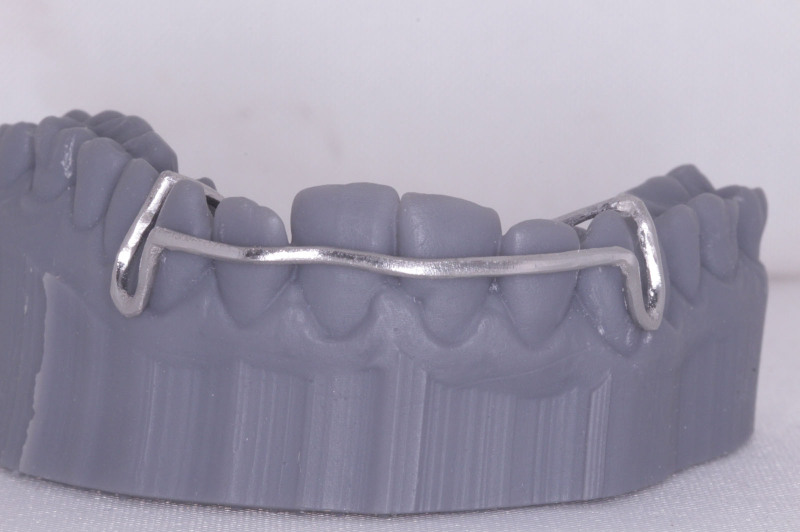
The fitting of the labial bow on 3D printed model.

The virtual alloy components were exported to selective laser melting (SLM) machine (SLM Realizer 2; Realizer GmbH, Lubeck, Germany) for fabrication using Co-Cr alloy (Mediloy S-Co, BEGO Bremer Goldschlägerei Wilh. Herbst GmbH & Co. KGWilhelm-Herbst-Str. 1 · 28359 Bremen, Germany). Supports were added by a semiautomatic process of the software, which was easily removed (Fig. 10). Finally, after removing the support structures and polishing, the final alloy components were ready to be electropolished (Fig. 11). Upon finishing the alloy components, the Adams clasps (Fig. 12) and the labial bow (Fig. 13) were fitted on the 3D printed models.

#### 2.6.2. Assembly of the Hawley retainer components

The base plate was inspected for irregularities or sharp edges. The PEEK base plate was trial-fitted on the 3D-printed model, and alloy components were inserted into the bespoke channels.

**Figure 14.**
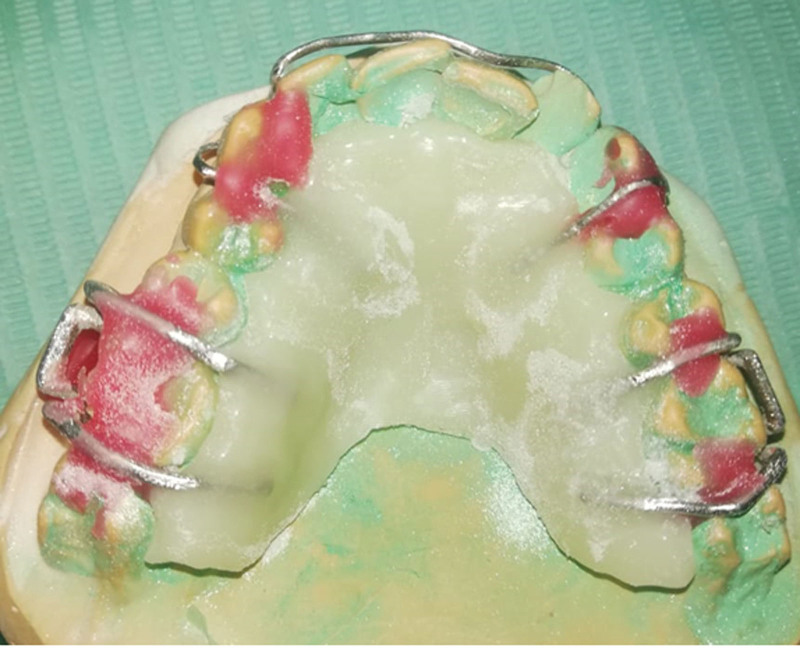
The application of cold-cured acrylic into bespoke channels for clasps assembly.

The alloy components were secured buccal to the teeth using baseplate wax while applying acrylic (Fig. 14). Cold-cured acrylic (ProBase Cold Acrylic Resin, Ivoclar Vivadent Inc.) was used to secure the clasp tags into the bespoke channels. A separating medium, Vaseline, was painted on the surface of the 3D-printed model to prevent clod-cured acrylic from adhering to the surface. The acrylic resin was added incrementally into the bespoke channels to ensure complete coverage of the clasp tags. The 3D model and device were placed in the pressure pot at 20 psi for 20 minutes to ensure polymerization.

Polishing burs commonly used in the dental laboratory were used to finish and polish the acrylic. Rubber burs were also used to polish alloy components to achieve a shiny luster.^[8]^ To assess the appliance fit on the 3D model, a spray fit checker (Quick Check Disclosure Indicator Spray, Vacalon Company) was used. It was then tried in the case mouth to ensure accurate fit and comfort.

## 3. Results

### 3.1. Evaluation of the design and fabrication technique

This prototype proves the possibility of manufacturing removable orthodontic appliances from intraoral scans without making impressions using dual CAM techniques.^[9–13]^ Additive and subtractive technologies produced a milled PEEK baseplate and SLM-3DP alloy components. The novelty of this prototype demonstrates how data from an intraoral scan can be used to manufacture a removable appliance. Cold-cured acrylic resin was used to assemble the components, and it was deemed to be functional. The authors indicated that the favorable greater corrosion resistance of the direct metal laser sintering-built alloy has excellent potential for dental application, and the result of this study emphasizes their views.

### 3.2. Assessment of the fit of the appliance

The appliance was inspected for sharp blebs and irregularities. The tag ends of the alloy components were wholly embedded in the baseplate, and the appliance polished surface was utterly polished.

**Figure 15.**
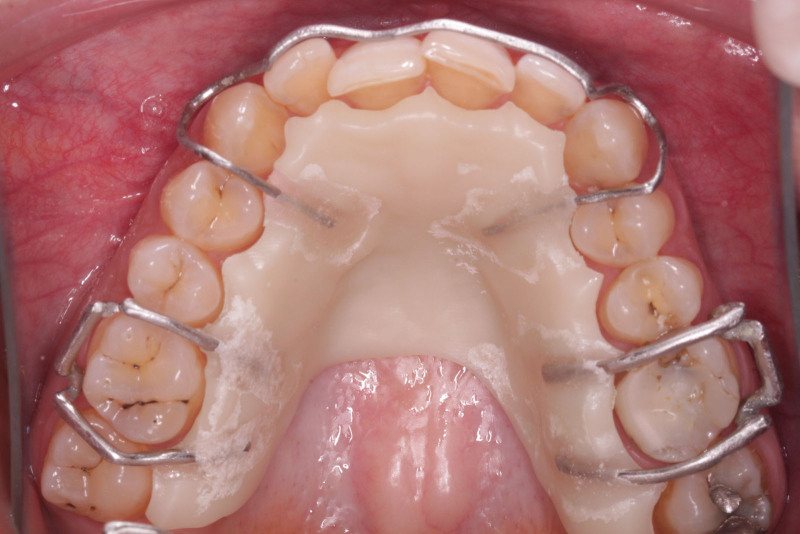
The fitting of the Hawley retainer intraorally.

**Figure 16.**
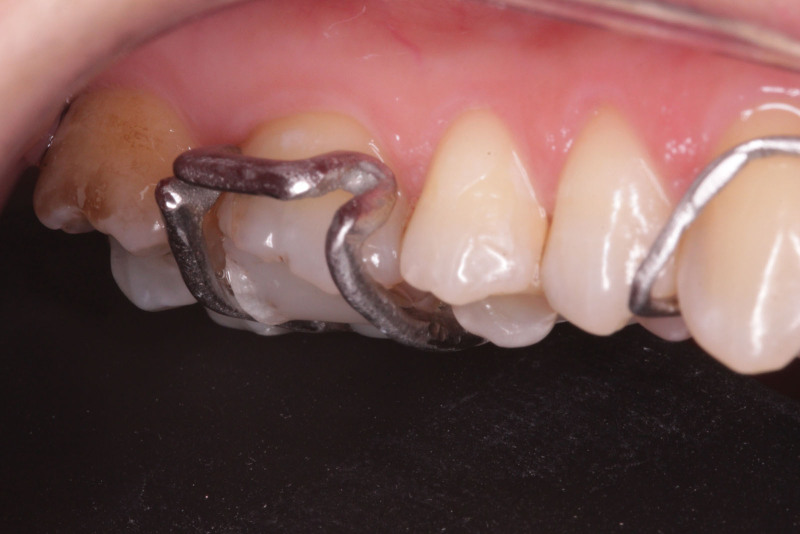
The arrowheads fitted into the undercuts.

The quality of intraoral fit was assessed.^[14]^ The appliance was seated fully around the teeth, the baseplate fitted closely against the palatal tissues, and the labial bow fit snugly against the teeth without any obvious air gaps between the wire and the teeth (Fig. 15). The arrowheads were fitted intraorally into the undercuts and were clear of the buccal mucosa and cheeks (Fig. 16). In addition, the patient was asked to reflect on the device comfort and fit, and instructions were given on inserting and removing the device from the mouth. The study protocol was approved by the IRB Committee at Jordan University of Science and Technology, and written informed consent was obtained from the study participants.

## 4. Discussion

When fabricating a removable orthodontic appliance with a fully digital dental workflow, a standard tessellation language file is generated using an intraoral scan, which the dentist uses to design and manufacture the prosthesis. This present report used a 3Shape intraoral scanner (TRIOS, Copenhagen, Denmark). It was reported that newer generations of intraoral scanners are sufficiently accurate for generating clinically acceptable complete arch digital impressions.^[15]^

The accuracy of an intraoral scan encompasses 2 definitions: trueness and precision.^[16]^ Trueness of an intraoral scan is defined as the deviation from the actual dimension object.^[17]^ While precision is defined as the measure of repeatability and how the results are close to each other.^[17]^

The manufacturing technique described in the study illustrates the feasibility of using digital technology entirely to build removable appliances. This was a development from a previous study, in which the Hawley retainer was produced in the CAD environment using Freeform software, where all components were manufactured by AM.^[18]^ In the digital workflow for designing removable appliances, the metal framework is designed using specialized dental CAD software such as EXOCAD. The design process includes the digital workflow in which the framework is designed, printed, or milled into a 3D resin pattern, which is then milled into Cr/Co alloy. The demonstrated technique requires special skills in nondental CAD software such as FreeForm or Meshmixer for the design.

The report describes the step-by-step procedure for designing and fabricating the PEEK baseplate and the wire Adams clasp. These steps could be used as a future reference to reproduce the results in the present report and potentially improve on them. Significant time and effort were invested in learning how to operate the software and hardware. The described method indicates that the digital interface requires the user to alter the design process to adapt to the advanced manufacturing technologies. One of the challenges in manufacturing the Hawley retainer was assembling the components using cold-cured acrylic, as attention was required to avoid adhering the acrylic to the 3D model. A suitable separating medium is necessary to use before the application of cold-cured acrylic to prevent adherence of the material to the surface of the 3D printed model.

Both PEEK and SLM alloy materials used in this study met the toxicity testing. They were known eventually as biocompatible materials and thus safe to be used intraorally for long periods. The alloys showed a safe level of elution according to the ISO definition in all investigated acidic environments, and the greatest elution occurred in the most acidic environment.^[18]^ SLM is considered to have excellent potential for dental applications and is suitable for the fabrication of orthodontic appliances, removable partial dentures, dental restorations, and implant prostheses.^[18]^ The elution of ions (Co, Cr, and Mo) in artificial saliva from the SLM alloy was lower than that from the cast alloy.^[18]^ The authors indicated this improved performance could be due to the addition of tungsten, which is known to improve the corrosion properties of Co-Cr alloys and to reduce chromium-depleted inter-metallic areas.^[18]^

The additively manufactured alloy metallurgical characteristics and physical properties differ significantly from those of conventionally produced wrought wire.^[19]^ SLM process allows control of the parameters; thus, different shapes and thicknesses of the alloy components are possible. A study of self-ligating appliance Co-Cr alloy physical characteristics, microstructure, and hardness indicates the possibility of replacing the conventional cast Co-Cr clasps.^[20–22]^ However, the clinical behavior of the material might differ, which needs to be explored in further detail. The SLM-3DP components described in this paper were sufficiently successful. Alloy components offered great rigidity, yet further studies are needed to modify the composition of the alloys to get the required flexibility similar to that of wrought wires.

The appropriate thickness of SLM Co-Cr alloy wires is still questionable. Thus, the resultant alloy components were designed to be thicker than the recommended dimensions of counterparts’ wrought wire to avoid clasp breakage and to permit finishing and polishing procedures. A larger diameter of clasps than the ultimately required is recommended to permit material removal for electropolishing.

There is a tendency to assess the 3DP dental appliances using engineering computation to permit the wide use of digital appliances. A finite element analysis study on a printable Hyrax appliance manufactured from Remanium Star (Dentaurum, Ispringen, Germany) by 3Shape software showed the printable Hyrax appliance withstands the stress generated during activation and is deemed positive and clinically efficient.^[1]^ Authors commented that the flexibility of their appliance design and the metal alloy biocompatibility and strength offer a huge potential for more advanced appliance design. The technique described in this paper aligns with their views, potentially simplifying clinical procedures and having an excellent impact on the future of digital dentistry.

This report proposes a novel design of bespoke channels in the PEEK baseplate for the addition of CAD Cr/Co claps. The novelty of this design lies in the possibility of using it to fabricate digital long-term provisional removable partial dentures or removable orthodontic retainers, which should be followed by a clinical trial to validate their clinical usability and clinical performance.

## 5. Conclusion

The technique described in this paper presents a digital design and manufacturing technique that is potentially an alternative to the conventional lab-based technique. The novel design could be extrapolated to other ventures, including removable orthodontic appliances and partial dentures. The successful application of additive and subtractive manufacturing technologies in the manufacturing of a removable appliance was confirmed by this work. Future research could focus on material testing to determine the most appropriate thickness of the alloy components and physical properties using various clasp designs and the device intraorally functional functionality.

## Author contributions

**Conceptualization:** Noor A. Al Mortadi, Lina Khasawneh, Karem H. Alzoubi.

**Data curation:** Noor A. Al Mortadi, Lina Khasawneh, Karem H. Alzoubi.

**Formal analysis:** Noor A. Al Mortadi, Lina Khasawneh.

**Investigation:** Noor A. Al Mortadi, Lina Khasawneh, Karem H. Alzoubi.

**Methodology:** Noor A. Al Mortadi, Lina Khasawneh.

**Project administration:** Noor A. Al Mortadi, Lina Khasawneh.

**Resources:** Noor A. Al Mortadi, Karem H. Alzoubi.

**Software:** Noor A. Al Mortadi.

**Supervision:** Noor A. Al Mortadi.

**Validation:** Noor A. Al Mortadi, Lina Khasawneh.

**Visualization:** Noor A. Al Mortadi, Lina Khasawneh.

**Writing – original draft:** Noor A. Al Mortadi, Lina Khasawneh.

**Writing – review & editing:** Noor A. Al Mortadi, Karem H. Alzoubi.
